# Data in support of in vivo studies of silk based gold nano-composite conduits for functional peripheral nerve regeneration

**DOI:** 10.1016/j.dib.2015.05.020

**Published:** 2015-06-16

**Authors:** Suradip Das, Manav Sharma, Dhiren Saharia, Kushal Konwar Sarma, Monalisa Goswami Sarma, Bibhuti Bhusan Borthakur, Utpal Bora

**Affiliations:** a Department of Biosciences and Bioengineering, Indian Institute of Technology Guwahati, Guwahati 781039, Assam, India; b Saharia׳s Path Lab & Blood Bank, Guwahati 781005, Assam, India; c Department of Surgery and Radiology, College of Veterinary Sciences, Khanapara, Guwahati 781022, Assam, India; d Nemcare Hospital, Guwahati 781005, Assam, India; e Department of Surgical Oncology, Dr. B. Borooah Cancer Institute, Gopinathnagar, Guwahati 781016, Assam, India; f Mugagen Laboratories Private Limited, Technology Incubation Centre, Indian Institute of Technology Guwahati, Guwahati 781039, Assam, India

## Abstract

In the present data article we report the in vitro and in vivo biocompatibility of fabricated nerve conduits described in Das et al. [1]. Green synthesised gold nanoparticles (GNPs) were evaluated for their cytotoxicity in rat Schwann cells (SCTM41). We also describe herein the adhesion and proliferation of Schwann cells over the nanofibrous scaffolds. Methods describing surgical implantation of conduits in a rat sciatic nerve injury model, confirming its accurate implantation as well as the porosity and swelling tendency of the nerve conduits are illustrated in the various figures and graphs.

## Specifications table

**Table:** 

Subject area	Biology, Chemistry
More specific subject area	Nerve regeneration, nerve conduit
Type of data	Image (MRI, microscopy), graph, figure
How data was acquired	• Cytotoxicity values were acquired through UV–vis multiwell plate reader (Make-TECAN). • Live–dead staining results were acquired through inverted fluorescence microscope (Make-ProSciTech), • Images of nanofibers and cell-loaded nanofibers were acquired through FESEM (Make-ZEISS, Model-Sigma), • MRI images taken using 1.5T MRI machine ((Make-Philips Model-Achieva). • Electrical resistance of scaffolds were measured through Sub-femto-ampmeter (Make-Keithley).
Data format	Raw data (digital photographs), Analyzed (Image J software, NIH, USA)
Experimental factors	• Nanofibers and nerve conduits were sterilised by methanol and UV prior to cell culture and in vivo intra-dermal test respectively. • Nanofibers with cells were fixed with 4% paraformaldehyde prior to FESEM.
Experimental features	• MTT assay, live–dead staining. • FESEM of nanofibers and cell growth over nanofibrous scaffolds. • Electrical conductivity of nanofibrous scaffolds. • Intracutaneous irritation and toxicity study of nerve conduits as per ASTM STP 1452. • Surgical method of implantation of nerve conduits. • Magnetic resonance Imaging of implanted nerve conduits. • Porosity and swelling characteristics of nerve conduits.
Data source location	N/A
Data accessibility	All the data are embedded in the article.

## Value of the data

• In vitro and in vivo toxicity study of green synthesised gold nanoparticles and nanocomposite based nerve conduits respectively provides an insight into the potential safety of using nanoparticle based neural implants.
• Method for culturing and monitoring cellular proliferation over nanofibrous scaffolds.
• Data for porosity and swelling ratio of the conduits can be compared to other three dimensional scaffolds.

## 1 Data, experimental design, materials and methods

Green synthesised gold nanoparticles (GNPs) were evaluated for their cytotoxicity in rat Schwann cells (SCTM41). We also describe herein the adhesion and proliferation of Schwann cells over the nanofibrous scaffolds. Data supporting methods to surgically implant conduits in a rat sciatic nerve injury model, confirming its accurate implantation as well as the porosity and swelling tendency of the nerve conduits are illustrated in the various figures and graphs.

### 1.1 Cytotoxicity of synthesized GNPs

Cytotoxic effect of *Centella asiatica* mediated synthesized gold nanoparticles (GNP) was assessed by MTT assay on rat schwann cell line (SCTM41) following standard assay procedures [2]. Briefly, the nano-particle solution was lyophilized to get GNP as powder form. A stock solution (10 mg/ml) was prepared in Dulbecco׳s modified eagle׳s medium (DMEM) without serum. This solution was used to make further working concentrations (5 µg/100 µl to 500 µg/100 µl) of GNP against which MTT assay was done. The cells were cultured in 96 well cell culture plates and incubated with various concentrations of GNP (5 µg/100 µl to 500 µg/100 µl) in serum free DMEM for 24 h in a CO_2_ incubator (Make – Healforce) under 37 °C and relative humidity 90%. This was followed by addition of DMSO and recording absorbance at 570 nm using a UV–vis multiwell plate reader (Make-TECAN).

**Fig. 1:**
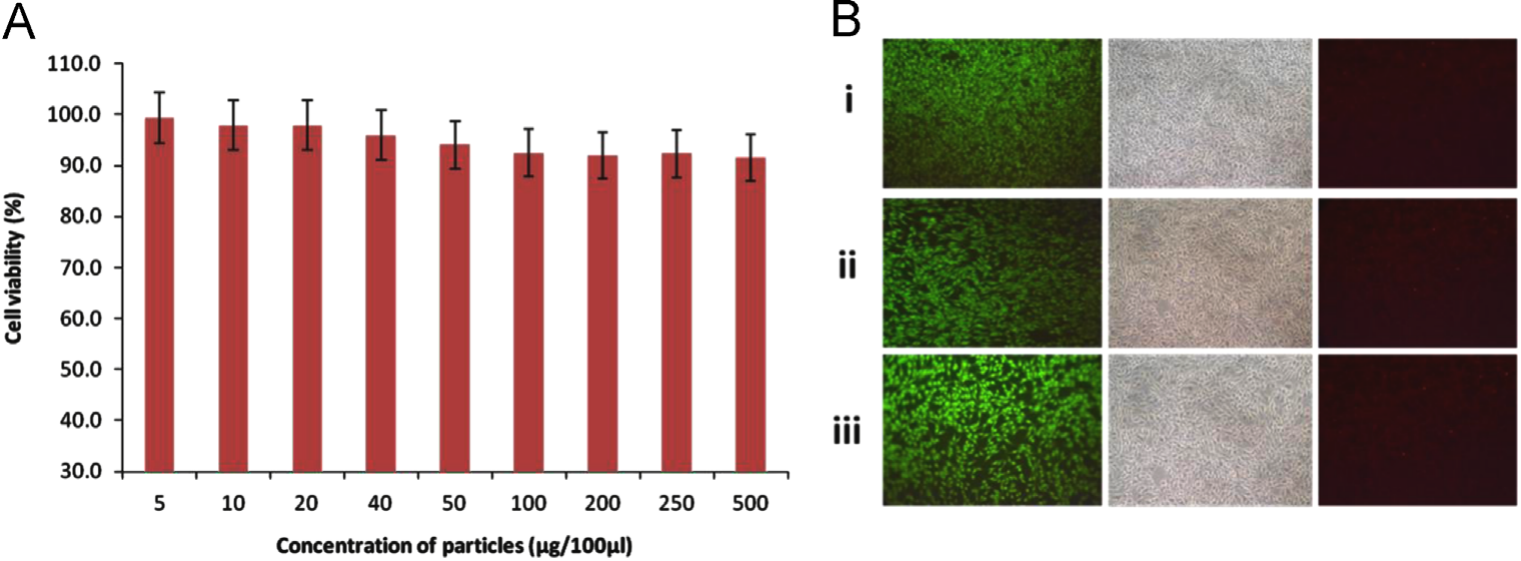
Cytotoxicity of GNPs. (A) MTT assay of GNPs on rat Schwann cell line SCTM41. Each concentration was done in triplicate to generate standard deviation. (B) Live–dead staining assay with Acridine orange (AO) and Ethidium bromide (EtBr).The assay was done by treating the cells with GNP concentration of 5 μg/100 μl (i), 50 μg/100 μl (ii) and 500 μg/100 μl (iii). The live cells are stained green from AO while the dead cells stain red with EtBr.

The gold nanoparticles synthesised using ethanolic extract of *C. asiatica* were found to be non-toxic to SCTM41 rat schwann cells up to a concentration of 500 µg/ml (Fig. 1A). Live dead assay with Acridine orange and Ethidium bromide conducted with GNP concentration of 5 µg/ml (Fig. 1B-i), 50 µg/ml (Fig. 1B-ii) and 500 µg/ml (Fig. 1B-iii) revealed normal cellular morphology up to the highest concentration of 500 µg/ml. The live cells preferentially take up Acridine orange (green) whereas Ethidium bromide (red) stains the nuclei of dead cells [3].

### 1.2 Electrical resistance of scaffolds

**Fig. 2:**
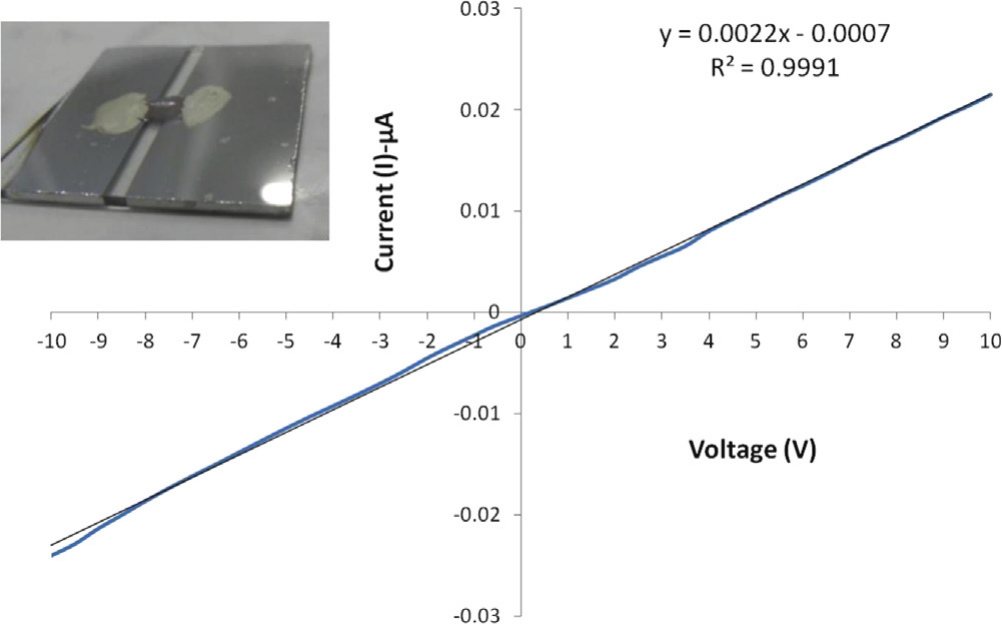
Electrical property of GNP-SF nanocomposite scaffold. The figure shows the ohmic nature of the *I*–*V* characteristic of the GNP-SF nanocomposite scaffold with the experimental setup in inset.

A piece of corning glass was first coated with aluminium using a suitable masking agent to generate a 2 mm thick uncoated strip in the middle. In order to study the electrical resistance, a 1 cm×1 cm portion of the electrospun silk fibroin (SF) and gold nanoparticle-silk fibroin nanocomposite (GNP-SF) sheet was placed over the uncoated strip with its edges fixed on the aluminum coated section by silver paste. An *I*–*V* graph was generated for GNP-SF sample (Fig. 2) and the resistance of the materials was calculated.

### 1.3 Architecture of nanofibers and culture of Schwann cells over nanofibrous scaffolds

**Fig. 3:**
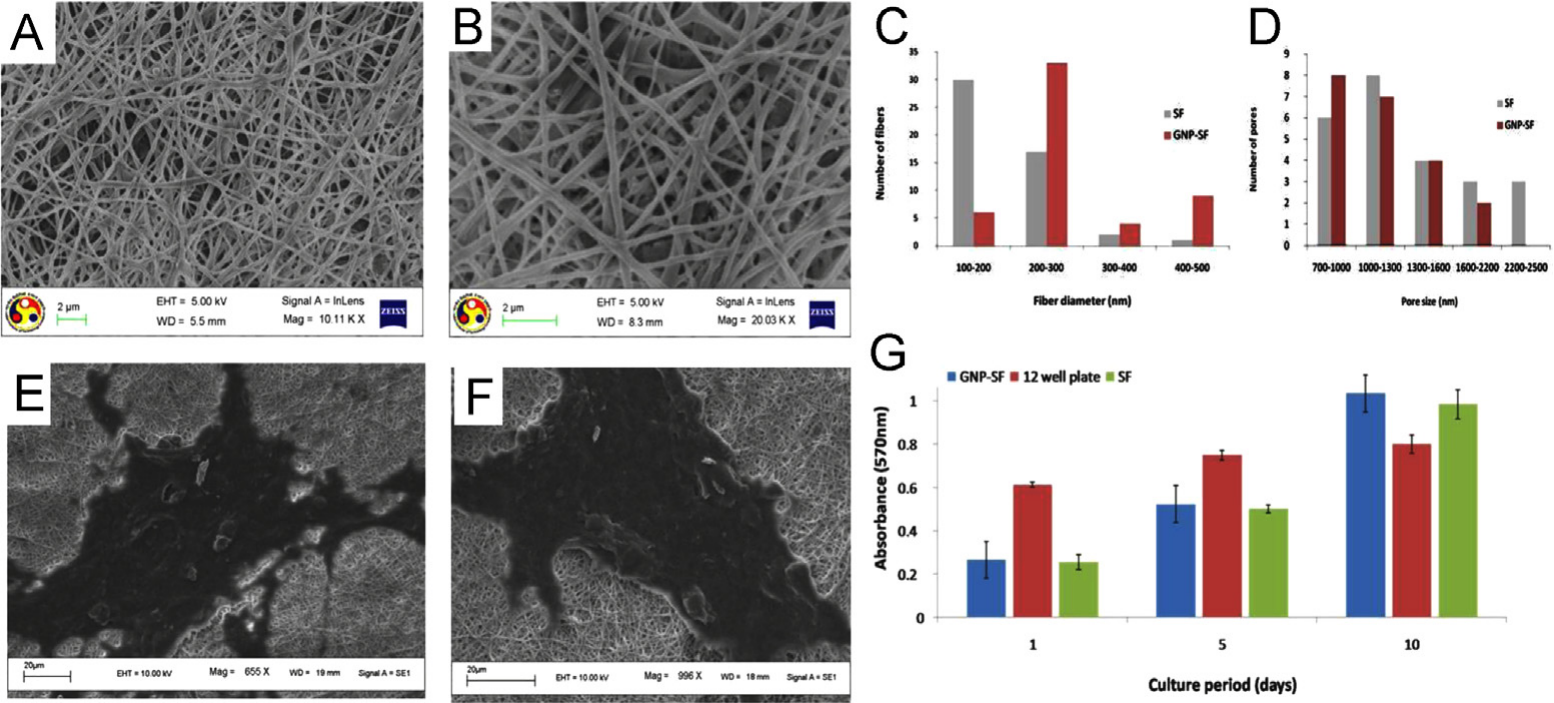
Architecture of nanofibers and culture of Schwann cells over nanofibrous scaffolds. (A) FESEM image of SF nanofibrous scaffold and (B) nanocomposite (GNP-SF) nanofibrous scaffold. (C) Size distribution of nanofibers calculated using Image J (NIH, USA), (D) pore size distribution of the nanofibrous scaffolds calculated using Image J (NIH, USA), (E) FESEM image of SF nanofibrous scaffold after 5 days of culturing Schwann cells (SCTM41) and (F) FESEM image of GNP-SF nanocomposite nanofibrous scaffold after 5 days of culturing Schwann cells (SCTM41). (G) Cell proliferation study by MTT assay by culturing Schwann cells for 10 days over GNP-SF and SF scaffolds keeping one well of 12-well culture plate as control.

FESEM analysis of the electrospun mats (both GNP-SF and SF) showed the nanofibers were distributed in a mesh like architecture (Fig. 3A and B). Pore size distribution and fiber diameter of the nanofibers were studied using Image J. The nanocomposite nanofibers were observed to have a larger diameter of 200–300 nm and smaller pore size distribution in the range of 700–1000 nm as compared to silk fibroin nanofibers (Fig. 3C and D). Pristine silk fibroin and GNP incorporated silk fibroin nanofibers were collected in cover slip separately for 2D culture of cells. Cover slips were treated with methanol and UV ray for conversion to *β* sheet and sterilization. They were then washed 3–4 times with sterile phosphate buffer saline (PBS) and incubated with DMEM containing 10% FBS for 24 h in CO_2_ incubator for conditioning. SCTM41 cells (rat Schwann cell) were seeded over cover slips and incubated for 10 days. On fifth day cover slips were processed for FESEM analysis to monitor cell adhesion. Cellular proliferation was also quantitatively studied over the cover slips by MTT analysis at 0, 5th and 10th day.

The cells were found to adhere and grow over the surface of electrospun nanofibers (Fig. 3A, B, E and F). MTT assay of cells growing over the scaffolds exhibited subsequent increment of absorbance with time over a period of 10 days as compared to a more saturated growth in control group of 12 well tissue culture plate (Fig. 3G). These results indicate enhanced cellular proliferation over porous nano-scaffolds with higher surface area to volume ratio than conventional tissue culture plates with similar dimensions. However, no appreciable difference was observed in rate of proliferation between GNP-SF and SF scaffolds.

### 1.4 In vivo intradermal test

**Fig. 4:**
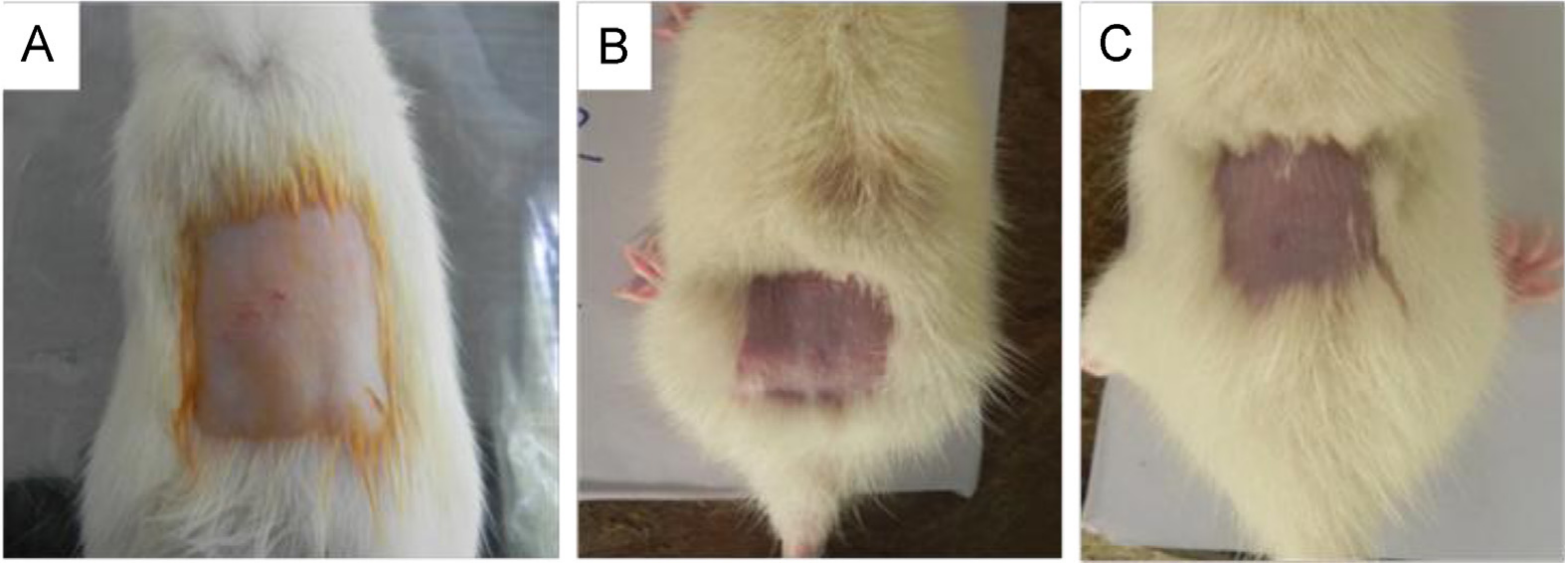
In vivo intracutaneous irritation test. The animals observed after 72 h of injecting saline extract of (A) GNP-SF conduit and (B) SF conduit. (C) In the control group only saline was injected.

Intra-cutaneous irritation and toxicity studies were performed on Sprague Dawley rats (*n*=9) following standard procedures as described in ASTM standard protocol for “Standards Used in Meeting Requirements for a Model Pre-Market Approval (PMA) of a Neural Guidance Conduit” (ASTM STP 1452). Briefly, 24 h saline extract of the silk fibroin (*n*=3) and GNP-incorporated silk fibroin conduit (*n*=3) were taken and injected intradermally. Normal saline was injected in the control group (*n*=3) (Fig. 4C). The animals were observed for 72 h.

The 24 h saline extract of the GNP-SF (Fig. 4A) and SF conduits (Fig. 4B) did not produce any abnormal clinical symptoms, oedema or erythema in the animals. The site of administration of the extract did not exhibit any abnormality as compared to the control saline group up to 72 h from the time of intra-dermal injection.

### 1.5 Surgical implantation and electrophysiological study

**Fig. 5:**
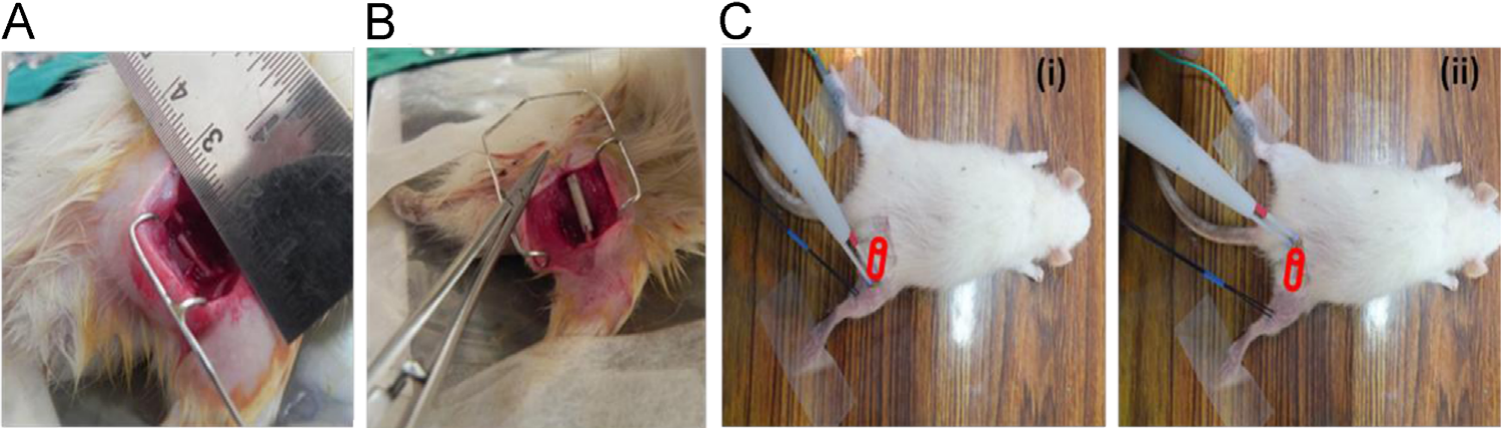
Surgical implantation and electrophysiological study. (A) Rat sciatic injury model having a 10 mm gap was created. (B) The fabricated nerve conduits were implanted within the gap and sutured to the proximal and distal ends of the nerve stump. (C) Experimental setup for conducting NCV and CMAP studies with the nerve stimulator (white) and recording electrode (black). The nerve stimulator is placed once at the distal end (i) and again at the proximal end (ii) of the implanted conduit (apparent position shown in red) to record NCV through the conduit. The recording electrode is kept fixed near the ankle of the animal.

A 10 mm gap was created in the sciatic nerve and the conduits were implanted and secured to the proximal and distal ends of the nerve by suture (Fig. 5A–B). For the electrophysiological study of measuring nerve conduction velocity (NCV) and compound muscle action potential (CMAP) the proximal and distal ends of the conduit were sequentially stimulated while the recording electrode was fixed near the ankle region of the animal (Fig. 5C)

### 1.6 Magnetic resonance imaging (MRI)

**Fig. 6:**
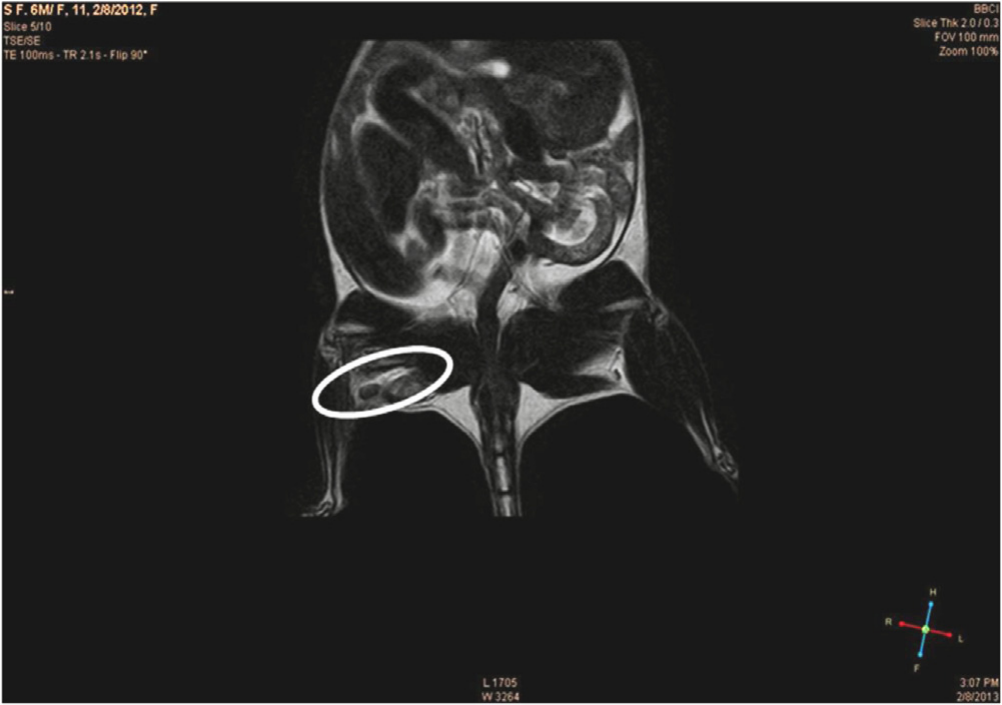
Magnetic resonance imaging of animals. The implanted conduit could be visualized (encircled area) by MRI after a week of implantation.

The animals were subjected to MRI imaging using a 1.5T MRI machine (Make-Philips Model-Achieva) and a 40 mm diameter coil one week post implantation. The conduit was found to be accurately placed in the sciatic nerve (Fig. 6).

### 1.7 Porosity and swelling ratio of nerve conduits

The porosity of the conduits was determined by a previously described ethanol displacement method [4]. Briefly, the dimensions of the conduits (*n*=4) were precisely measured using a vernier caliper and the volume (*V*) calculated according to the following formula:

$$V=\pi \left(R^{2}-r^{2}\right)h,$$

where *R* was the outer radius of the conduit, *r* was the inner radius of the conduit and *h* was the length of the conduit. The conduits were then weighed in dry condition (*M*_*o*_) and immersed in ethanol for 24 h before being weighed again to get *M*_*t*_. The porosity of the conduit was calculated according to the following formula:

$$Porosity\;\left(\%\right)=\;\frac{\left(M_{t}\right)-\;\left(M_{o}\right)}{V\times \rho }\times 100$$

where *ρ* is the density of ethanol (0.79 g/cc). Both SF and GNP-SF conduits were found to have low porosity. The porosity of SF nerve conduit was found to be 15.6% (±0.3) while GNP-SF conduits exhibited minute porosity of 5.37% (±0.08).

**Fig. 7:**
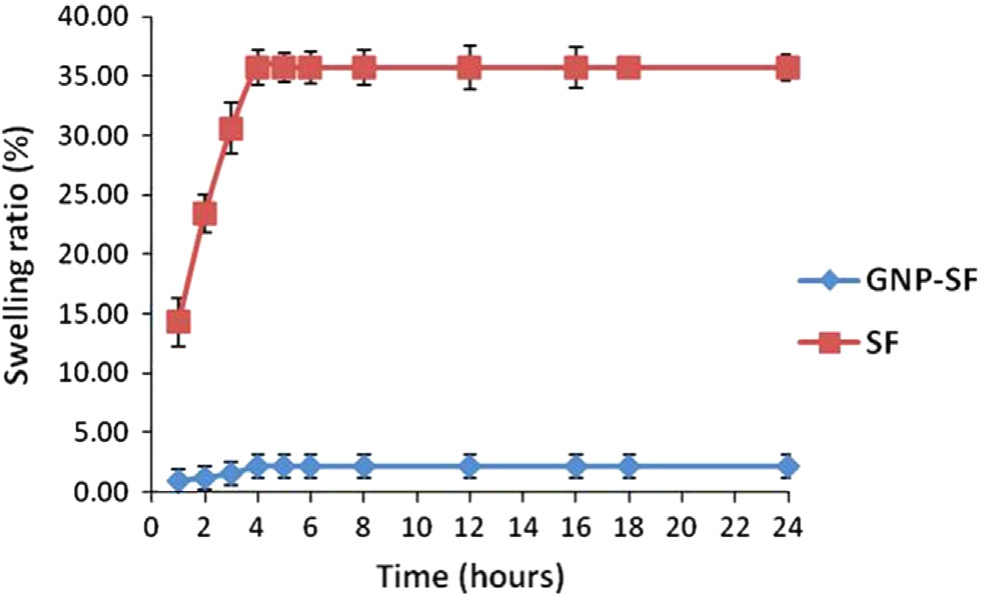
Swelling ratio of conduits. The dynamic swelling ratio of the conduits were calculated periodically every hour upto 24 h.

The dynamic swelling ratio of the nerve conduits was measured according to a previously described method [5]. The conduits were immersed in phosphate buffered saline (pH 7.4) and the swelling ratio was calculated periodically every hour according to the following formula:

$$\mathrm{Swelling}\;\text{ratio}\;\left(\%\right)=\frac{\left(W_{s}-W_{d}\right)}{W_{d}}\times 100$$

where *W*_*s*_ is weight of the conduit in swollen state and *W*_*d*_ is the dry weight. The SF conduits saturated at a swelling ratio of 35.7% while GNP-SF conduits showed a maximum swelling of only 2.1% (Fig. 7).

The low porosity and minimum swelling tendency of both GNP-SF and SF conduits can be attributed to the stacking of multiple layers of nanofibers during conduit fabrication [1].
